# Orofacial findings in chronic granulomatous disease: report of twelve patients and review of the literature

**DOI:** 10.1186/1756-0500-3-37

**Published:** 2010-02-17

**Authors:** Najla S Dar-Odeh, Wail A Hayajneh, Osama A Abu-Hammad, Huda M Hammad, Adel M Al-Wahadneh, Najwa K Bulos, Azmi M Mahafzah, Maha S Shomaf, Mohammed A El-Maaytah, Faris G Bakri

**Affiliations:** 1 Faculty of Dentistry, University of Jordan, Amman, Jordan; 2 Department of Pediatrics, Jordan University of Science and Technology, Irbid, Jordan; 3 Department of Oral Medicine and Surgery, Jordan University of Science and Technology, Irbid, Jordan; 4 Department of Pediatrics, King Hussein Medical Centre, Amman, Jordan; 5 Department of Pediatrics, Jordan University Hospital, Amman, Jordan; 6 Department of Pathology and Microbiology, Jordan University Hospital. Amman, Jordan; 7 Division of Oral & Maxillofacial Medical, Surgical & Diagnostic Services, UCL Eastman Dental Institute, London, UK; 8 Department of Internal Medicine, Jordan University Hospital, Amman, Jordan

## Abstract

**Background:**

Chronic granulomatous disease is an extremely rare primary immunodeficiency syndrome that can be associated with various oral complications. This can affect high number of patients. However, data on oral complications is sparse. Here we will review the literature and describe the orofacial findings in 12 patients.

**Findings:**

The age range was 5-31 years. Oral findings were variable, and reflected a low level of oral hygiene. They included periodontitis, rampant caries, gingivitis, aphthous-like ulcers, and geographic tongue. One patient had white patches on the buccal mucosa similar to lichen planus. Another patient had a nodular dorsum of the tongue associated with fissured and geographic tongue. Biopsies from the latter two lesions revealed chronic non-specific mucositis. Panoramic radiographs showed extensive periodontitis in one patient and periapical lesions in another patient.

**Conclusion:**

Patients with chronic granulomatous disease may develop oral lesions reflecting susceptibility to infections and inflammation. It is also possible that social and genetic factors may influence the development of this complication. Therefore, oral hygiene must be kept at an optimum level to prevent infections that can be difficult to manage.

## Background

Chronic granulomatous disease (CGD) is an extremely rare congenital immune deficiency syndrome with an incidence of 1:250,000 individuals. It is characterized by recurrent severe infections due to the inability of neutrophils and macrophages to mount a respiratory burst and kill invading bacteria and fungi. The disease is caused by nicotinamide adenine dinucleotide phosphate (NADPH) oxidase deficiency which results from mutation in one of the four components of the NADPH oxidase complex. The most frequent form is the X linked (XCGD), with mutations in *CYBB *gene encoding gp91*phox *subunit. Rare subgroups are caused by mutations in *CYBA*, *NCF1 *or *NCF2 *genes encoding p22*phox*, p47*phox *or p67*phox *subunits respectively [1,2]

CGD patients usually present in the first few years of life with cervical or inguinal lymphadenitis, liver abscesses, osteomyelitis, pneumonia, or skin infections [1,2]. In addition, CGD patients also suffer from infections and sterile hyperinflammation in the oral cavity [3]

In CGD, oral complications are generally considered to be minor infections as they are not life-threatening. Several reports describe oral ulcers [4-9], including one case with multiple buccal mucosal ulcers in direct contact with dental plaque [4]. Other findings include periodontal involvement such as severe gingivitis [4-6], periodontitis [4,6], generalized pre-pubertal periodontitis [10], granulomatous mucositis in the upper lip [11] and the soft palate [12], geographic tongue [9], oral candidiasis [13], and enamel hypoplasia [9]. However, it is not clear to what extent these complications can be attributed specifically to CGD since many of them are also common in the general population [3].

Histological findings of oral lesions have generally shown inflammation; Wysocki and Brooke [14] described an ulcerative lesion showing inflammatory cell infiltrate consisting of plasma cells, histiocytes, and occasional eosinophils. This is in addition to small granulomas characterized by mononuclear histiocytes and occasional multinucleated giant cells. One of the granulomas exhibited a central focus of necrosis containing moderate numbers of neutrophils and eosinophils. Another report by Dusi et al [11] described chronic inflammation and non-caseating granuloma composed of many epithelioid cells, a few giant cells, edema, and dilated lymphatic vessels in the superficial dermis in the biopsy of an upper lip granulomatous cheilitis. Furthermore, Cohen et al [6] also showed acute and non-specific inflammation from oral ulcers biopsies.

The frequency of oral complications in CGD is variable and can range from few cases in some large series [1,15,16], to as high as 35% in other studies [17]. Therefore, these complications could be a major cause of morbidity in patients with CGD. However, despite this occasional high frequency and potential morbidity, these complications have rarely been investigated [3].

Factors that predispose to CGD related oral complications include neutrophil dysfunction [18,19]; malnutrition due to gastrointestinal complications such as gastric outlet obstruction, colitis-enteritis, and diarrhoea [20,21]. Also included are autoimmune mechanisms [22]; and possibly immunosuppressive therapy, particularly steroids, which may be given to patients with granulomatous lung or inflammatory bowel disease [23]. Other factors for periodontal diseases that may occur in CGD patients, but are not specific to CGD, include smoking, poor oral hygiene, and emotional stress [23].

Here, we describe the oral findings in 12 CGD patients including the histological findings of oral lesions in two of them.

## Findings

Twelve CGD patients, from eight families, were referred from 3 major hospitals in Jordan. Patients A3, A4, D4, E8, and E10 suffered from a mutation in *NCF1*; C1 and F5 from a mutation in *NCF2*; J2 and J3 from a mutation in *CYBA*; B1 and B2 from an autosomal recessive type with unidentified mutation. Patient K1 was the only patient with the X linked form, he suffered from a rare X91^+ ^type. All patients were taking prophylactic itraconazole and trimethoprim-sulfamethoxazole, but on an intermittent basis. The full clinical and molecular characteristics of these patients were previously published [24]. Dental and medical histories, including drug intake, history of oral hygiene, and clinical oral examination were performed and documented for all patients. Panoramic radiographs, to detect any bony or dental pathology, were performed for all patients except patient B2 as she was uncooperative. Gingivitis index was measured and oral mucous membranes were examined for any abnormalities particularly ulcers. Specific oral lesions were seen in two patients, and these were subsequently biopsied.

### Orofacial findings

**A3**: Thirty-one year old man with grade 3 gingivitis, multiple carious teeth, badly distracted teeth, and submental salivary gland enlargement. Panoramic radiograph showed extensive periodontitis of the remaining roots apices.

**Figure 1:**
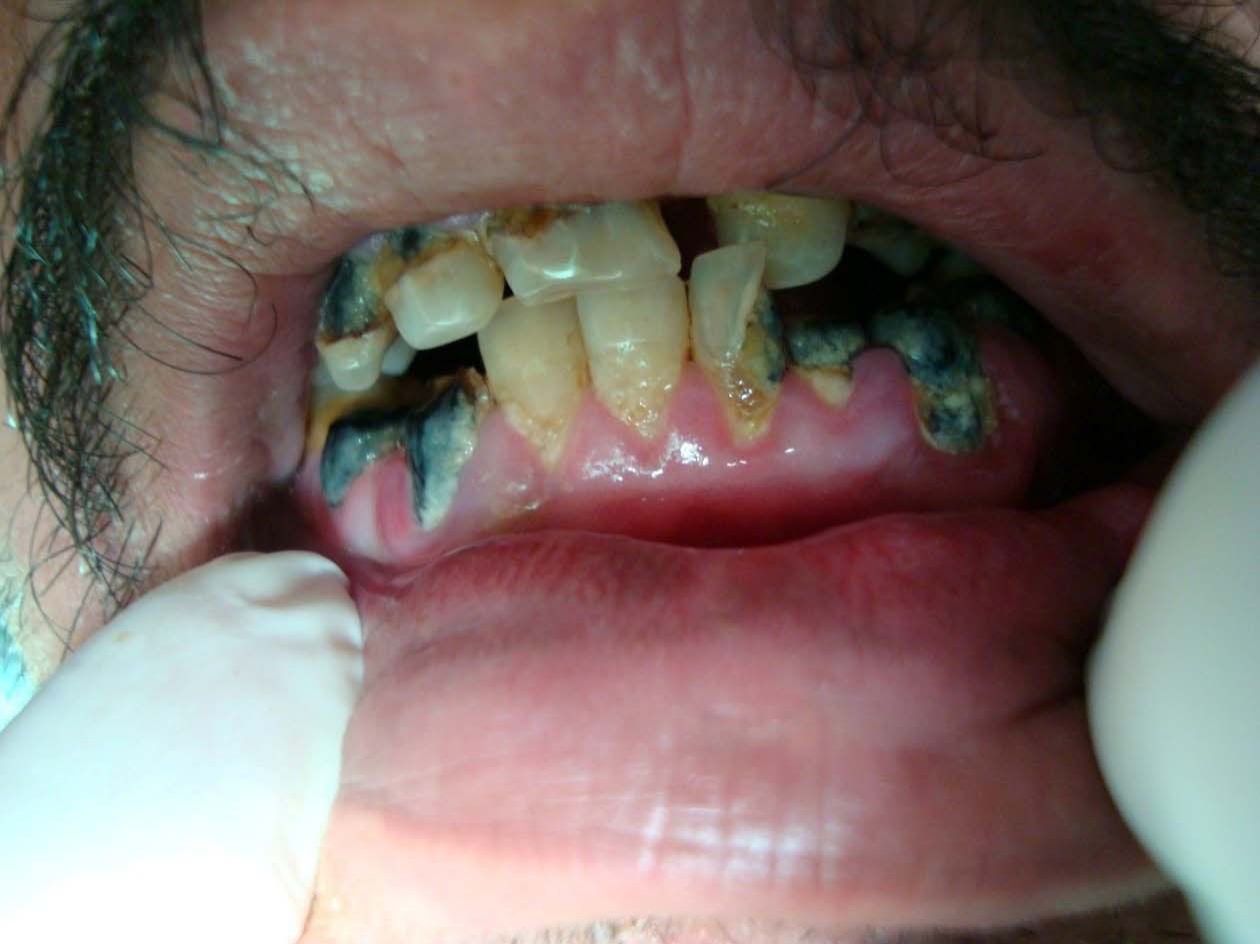
**Oral findings in patient A4**. Rampant caries and gingivitis.

**Figure 2:**
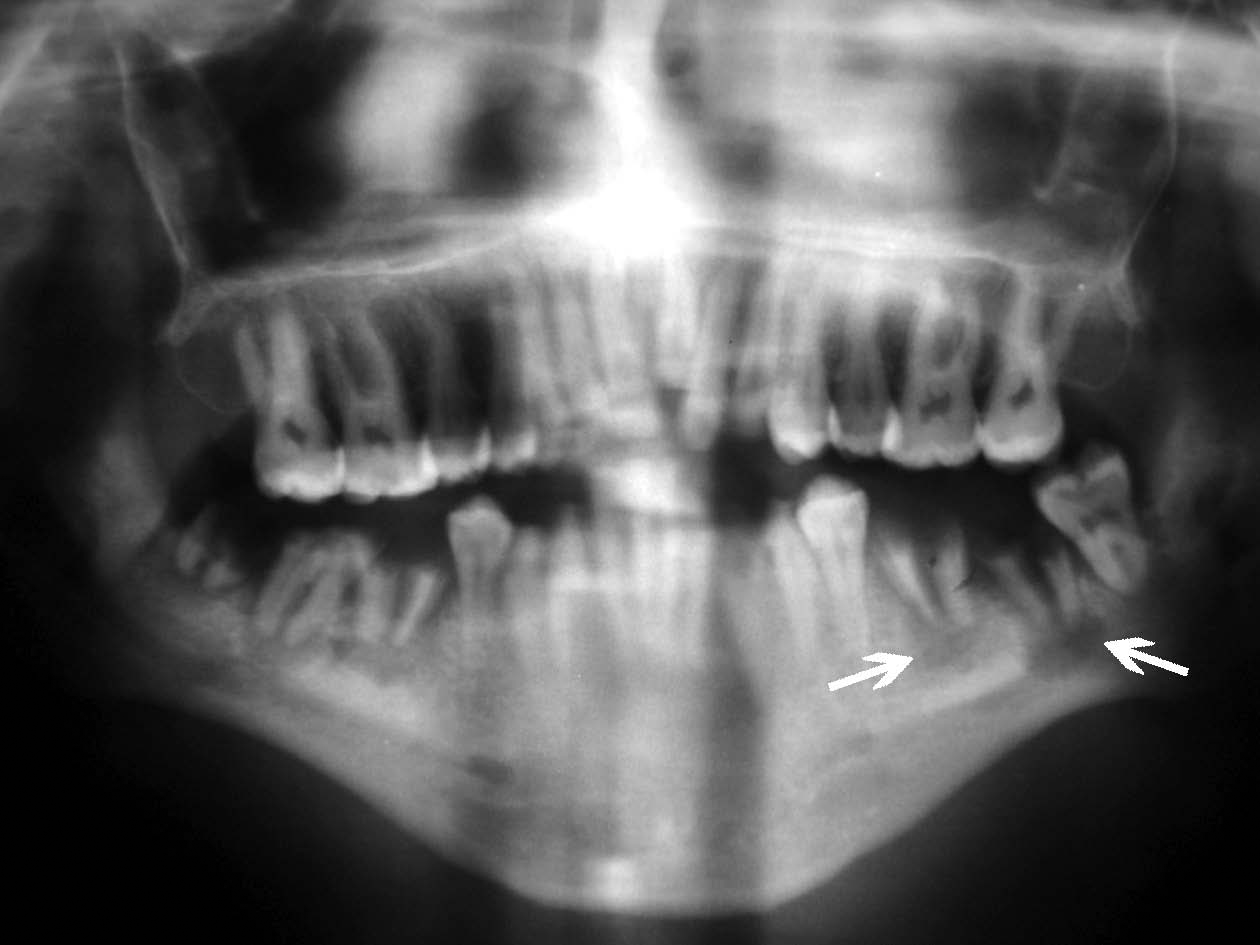
**Oral findings in patient A4**. A panoramic radiograph showing multiple periapical lesions related to lower molars (arrows).

**Figure 3:**
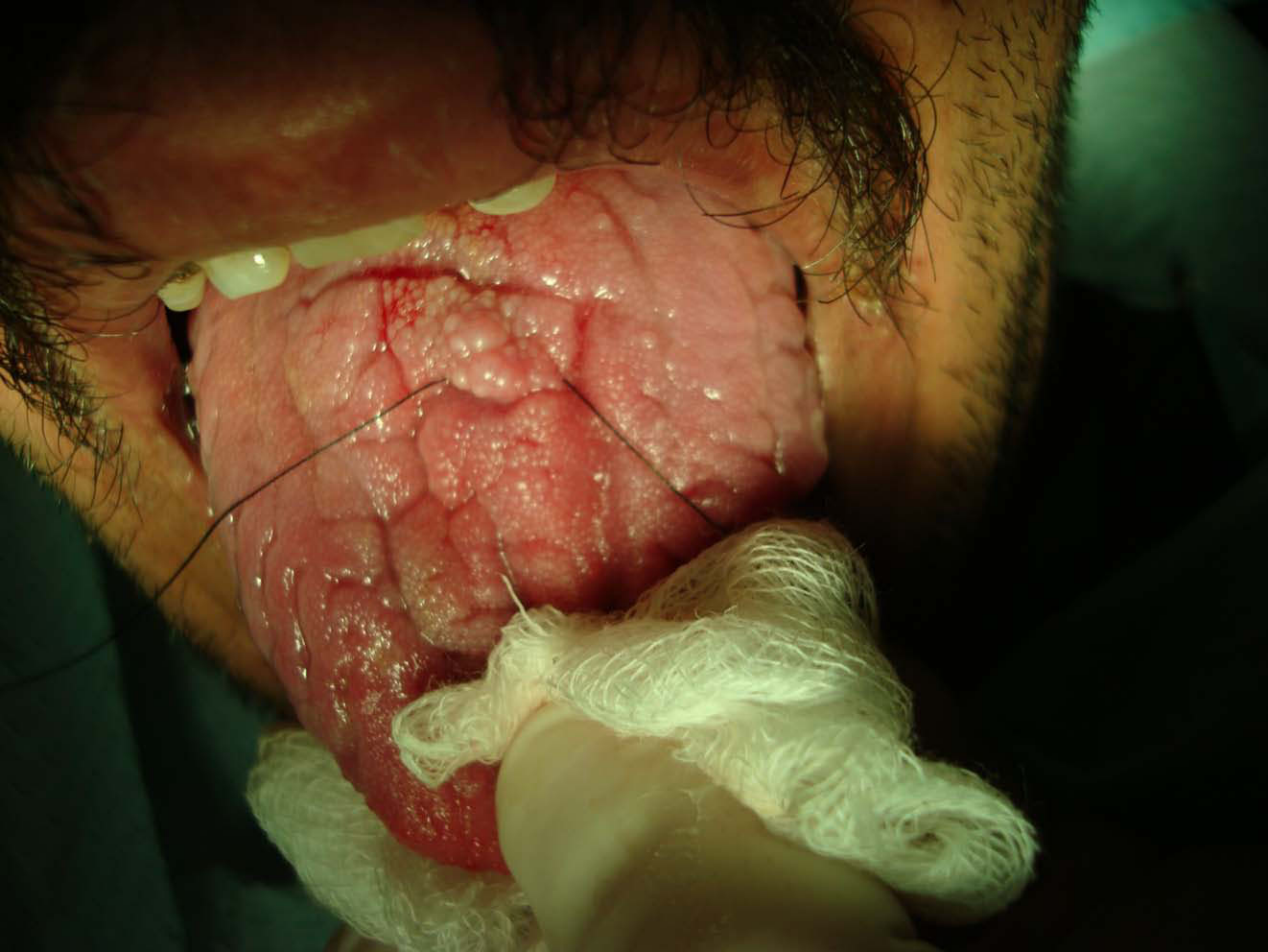
**Oral findings in patient A4**. Geographic tongue and the site of nodular lesion biopsy.

**Figure 4:**
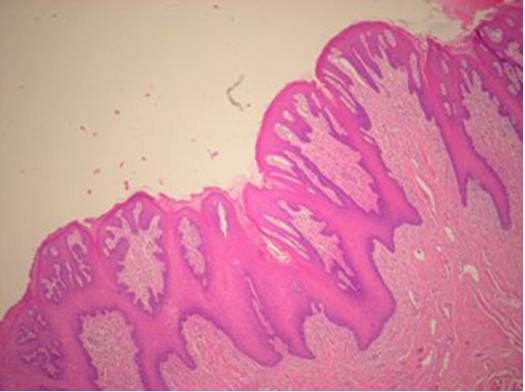
**Oral findings in patient A4**. Hyperkeratosis, acanthosis, and diffuse chronic nonspecific mucositis (H & E, ×40).

**A4**: Twenty-six year old man with grade 3 gingivitis, rampant caries, 4 missing teeth, periodontitis, geographic and fissured tongue, macroglossia, and smoker's melanosis (Figure 1). Multiple periapical lesions were observed in his panoramic radiograph related to badly decayed lower molar teeth (Figure 2), which were later extracted. A nodular lesion in the center of the anterior two thirds of the dorsum of the tongue was biopsied (figures 3 and 4).

**B1**: Thirteen year old boy with 3 carious deciduous teeth.

**B2**: Six year old girl, and sister of B1, with grade 1 gingivitis and rampant caries. She also had extrinsic staining on her teeth due to chronic intake of sugar-containing iron supplements.

**C1**: Fifteen year old boy with a coated tongue, aphthous-like ulcers and traumatic lip lacerations.

**D4**: Thirteen year old boy with grade 1 gingivitis and carious lower first molars.

**Figure 5:**
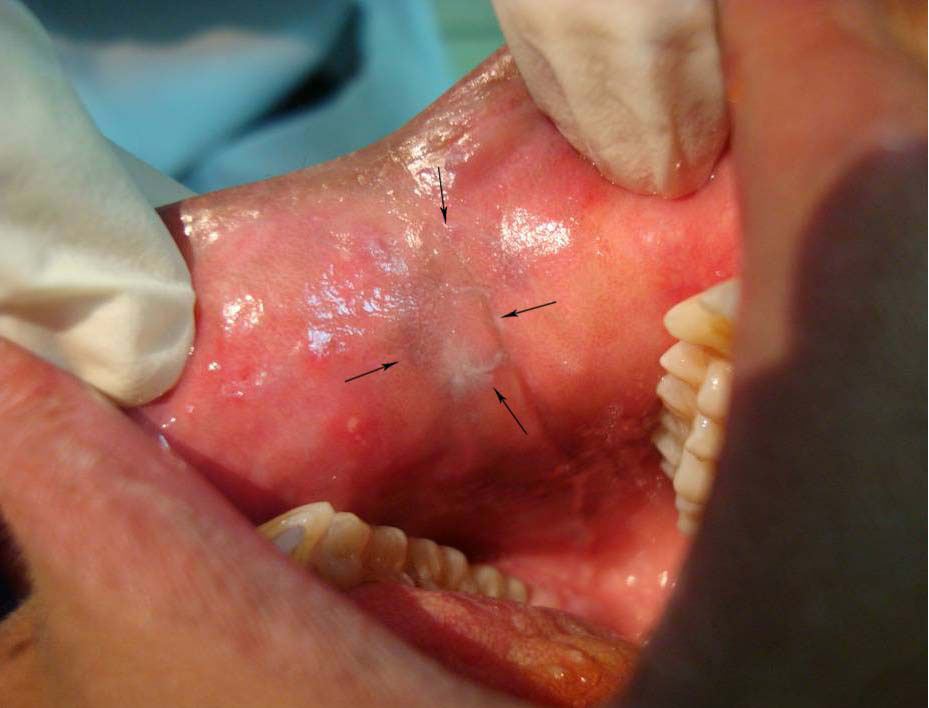
**Oral findings in patient E8 showing white lesions (arrows) in the form of thin striations affecting the buccal mucosa**.

**E8**: Fifteen year old boy with grade 1 gingivitis, fissured tongue. He also had characteristic white patches mimicking reticular lichen planus located on the buccal mucosa anteriorly and bilaterally, and on the junction between hard and soft palate (figure 5).

**E10**: Eleven year old boy, and brother of E8 with grade 1 gingivitis and 8 carious deciduous teeth.

**F5**: Seven year old girl with grade 1 gingivitis related to lower incisors.

**J2**: Seven year old girl with grade 3 gingivitis, rampant caries affecting 8 teeth and a geographic tongue. Five of her molars were in the form of remaining roots.

**J3**: Five year old girl, and sister of J2 with grade 3 gingivitis, 9 carious teeth and a geographic tongue. She had an abscess related to lower right molars with right submandibular lymphadenopathy.

**K1**: Fourteen year old boy with grade 3 gingivitis, facial scarring, and a facial wart.

### Histological findings

**A4**: An incisional biopsy of the nodular lesion on the center of anterior two thirds of the tongue showed hyperkeratosis, acanthosis, and mild diffuse chronic non-specific mucositis (Figure 4).

**E8**: The incisional biopsy taken from the white lesion in the right buccal mucosa showed mild hyperparakeratosis of surface epithelium, moderate fibrosis of the lamina propria with diffuse chronic non-specific mucositis, and mild chronic sialadenitis with occasional focal lymphocytic aggregates.

### Radiological findings

Panoramic radiographs did not show any specific lesions except in patient A3 and A4; A3 had extensive periodontitis of the remaining roots apices. A4 had multiple periapical lesions mainly due to neglect of oral hygiene with subsequent caries and pulpal involvement (Figure 2).

## Discussion

Patients in this study had variable oral manifestations. The severity of gingivitis and carious lesions was also variable. Unfortunately poor oral hygiene was observed in all patients in various degrees resulting in plaque-induced gingivitis and rampant caries (Figure 1). Six (50%) patients (A3, A4, B2, J2, J3, and E10) had rampant caries and multiple carious teeth. Periodontitis was observed in A3 and A4. Aphthous-like ulcers were observed in C1. Overall there were 7 (58%) patients suffering from significant oral complications. This is among the highest reported frequency [17]. However, accurate comparison to other reports is not possible due to: lack of detailed description of oral lesions in other reports, the small size of this series, and abundance of recessive type of CGD contrary to most reports where XCGD is the most common type. We also could not establish a relation between the severity of oral disease and CGD genotypes.

The oral health of the patients was neglected. The low level of oral hygiene and the inadequate use of a tooth brush may be attributed to the poor awareness of both the patients and their families to the importance of oral hygiene practices. Another cause seems to be the focus of parents on the general poor health of their children rather than on their oral hygiene.

Geographic tongue was observed in 3 (27%) patients (A4, J2, and J3), two of them who were sisters. However, although this association has been reported previously [9], it is unclear of it sigificance as this was a small sample, and the prevalence of geographic tongue in Jordan is approximately 7% [25].

In CGD, inflammation at multiple sites may lead to formation of exuberant tissue granulomas which can obstruct the larynx, oesophagus, or other regions of the gastrointestinal tract [26]. Biopsies of oral mucosa in our patients did not show granulomas. Rather, they showed non-specific mucositis with occasional lymphocytic aggregates. This is not surprising since CGD may present in a wide range of tissue specimens, most often demonstrating features of active chronic inflammation with or without granuloma formation [27].

The rate and severity of oral disease varies across different studies; one study [17] found rates of 35% while several other large studies [1,15,16,26,28,29] found low rates ranging from 0 to 26%. In our series we found a high rate of oral disease. This could be a specific finding in autosomal recessive types, or it could be due to factors other than CGD. These factors include neglect in oral hygiene, which was observed in all our patients. It is known that the most common factor for periodontal disease is the periodontal biofilm which forms on the teeth in the absence of effective oral hygiene [30]. Another factor is the possible genetic susceptibility related to the Jordanian study population as the prevalence of periodontitis varies between different races [31]. This is because genetic factors may play a role in periodontitis, the phenotype of which is determined by both the genetic makeup and the environmental influences on the patient [32]. Subsequently, our findings may not necessarily apply to CGD patients from other ethnicities and regions. Moreover, the abundance of autosomal recessive types of CGD here suggests the presence of co-segregated genes for periodontitis; similar to the previously described co-segregation of a neutrophil abnormality trait with localized aggressive periodontitis [33,34]. Furthermore, periodontitis has been reported with several rare genetic syndromes such as leukocyte adhesion deficiency, Ehler - Danlos syndrome type 8, and Lowe syndrome indicating the variety of genes that can predispose to periodontal disease [35].

Thirty years ago Wysocki and Brooke [14] noted that the oral manifestation of CGD was briefly alluded to, and variously described as, "ulcerative stomatits", "compatible with aphthous stomatitis", and "stomatitis". Today, this observation is still valid as most studies in CGD still refer to the oral complications with the same non specific terminology such as "gingivostomatitis" [17], "ulcerative stomatits", "gingivitis", and "periodentitis" [1,28,36] making it difficult both to make accurate comparisons between studies, and to describe the spectrum of the oral lesions. We therefore suggest that more specific terms be used instead to describe the oral manifestations in CGD.

## Conclusion

This study shows that CGD patients may have a high frequency and variable presentations of oral complications. Other social and genetic factors may influence the development of such complications. On a histological level, they may develop nonspecific chronic mucositis characterized by lymphocytic aggregation. The oral cavity can be a potential source of infection, especially if oral hygiene is neglected. Specialized dental care and regular dental attendance are crucial in the management of these patients.

## Competing interests

The authors declare that they have no competing interests.

## Authors' contributions

ND, designed the study, examined the patients, analyzed the data, and wrote the paper. OA, co-designed the study, participated in examining the patients, and in writing the paper. HMH, examined the patients. WAH, included the patients, and participated in writing the paper. NKB and AMA, included the patients in the study. MSS, performed the pathological reading on the biopsies. AM and MAE helped to draft the manuscript. FGB, conceived the study, supervised inclusion of patients, participated in both the study design, and the manuscript drafting. All authors read and approved the final manuscript.
